# Characterisation of tumor microenvironment and prevalence of CD274/PD-L1 genetic alterations difference in colorectal Cancer

**DOI:** 10.1186/s12885-023-10610-1

**Published:** 2023-03-09

**Authors:** Lin Yang, Shousheng Liu, Wenzhuo He, Zhenchong Xiong, Liangping Xia

**Affiliations:** 1 Department of Radiation Oncology, Nanfang Hospital, Southern Medical University, 1838 Baiyun Avenue North, Guangzhou, 510515 China; 2 State Key Laboratory of Oncology in South China, Collaborative Innovation Centre for Cancer Medicine, Sun Yat-sen University Cancer Center, 651 Dongfeng Road east, Guangzhou, 510060 China; 3 Department of General Medicine, Sun Yat-sen University Cancer Center, 651 Dongfeng Road east, Guangzhou, 510060 China; 4 Department of Breast Oncology, Sun Yat-sen University Cancer Center, 651 Dongfeng Road east, Guangzhou, 510060 China

**Keywords:** Fluorescence in situ hybridization, PD-L1, Mismatch repair deficiency, Immune microenvironment, Prognosis

## Abstract

**Background:**

Large-scale genomic alterations, especially CD274/PD-L1 gene amplification, have great impact on anti-PD-1 efficacy on cancers such as Hodgkin’s lymphoma. However, the prevalence of PD-L1 genetic alterations in colorectal cancer (CRC) and its correlation with the tumor immune microenvironment and clinical implications remain unknown.

**Materials and Methods:**

PD-L1 genetic alterations were evaluated in 324 patients with newly diagnosed CRC including 160 mismatch repair-deficient (dMMR) patients and 164 mismatch repair-proficient (pMMR) patients using fluorescence in situ hybridization (FISH) method. The correlation between PD-L1 and the expression of the common immune markers was analyzed.

**Results:**

Totally 33 (10.2%) patients were identified with aberrant PD-L1 genetic alternations including deletion (2.2%), polysomy (4.9%), and amplification (3.1%); They had more aggressive features such as advanced stage (P = 0.02), shorter overall survival (OS) (P < 0.001) than patients with disomy. The aberrations correlated with positive lymph node (PLN) (p = 0.001), PD-L1 expression by immunohistochemistry (IHC) in tumor cells (TCs) or tumor-infiltrated immunocytes (ICs) (both p < 0.001), and pMMR (p = 0.029). When dMMR and pMMR were analyzed independently, the correlations of aberrant PD-L1 genetic alterations with PD-1 expression (p = 0.016), CD4 + T cells (p = 0.032), CD8 T + cells (p = 0.032) and CD68 + cells (p = 0.04) were only found in dMMR cohort.

**Conclusions:**

The prevalence of PD-L1 genetic alterations was relatively low in CRC, but the aberrations usually correlate with aggressive nature. The correlation between PD-L1 genetic alterations and tumor immune features was only observed in dMMR CRC.

**Supplementary Information:**

The online version contains supplementary material available at 10.1186/s12885-023-10610-1.

## Introduction

Although considerable progress in the treatment of advanced colorectal cancer (CRC) has been made in the past 5 years, a substantial proportion of CRC patients still develop local recurrence and distant metastasis [1]. Immunotherapy has been on center stage for advanced cancer treatment since the groundbreaking success of immune checkpoint inhibitors (ICIs) in metastasized malignant melanoma [2]. The PD-1 ligands (PD-L1 or PD-L2) expression on the membrane of tumor cells or immune cells is a significant but not a definitive prediction indicator of the response to PD-1 blockade in some cancers [3–7]. *Patel et al.* raised doubts about this standard since patients’ void of immunohistochemically detectable PD-L1 expression still responded to inhibition of PD-L1 [8]. It has also been reported that early reduction in PD-L1 expression predicts faster treatment response in human cutaneous leishmaniasis[9]. Currently, there is still no agreement on the interpretation and criteria of PD-L1 expression and therapy response. Potentially, identification of CRC with effective molecular biomarkers could be helpful for determining prognosis.

Since the original identification on Hodgkin’s lymphoma[10, 11], PD-L1 gene amplification has been described in many tumors, such as triple negative breast cancer[12–14], a subset of EBV-positive gastric cancer [10, 15], lung cancer[16, 17], and diffuse large B-cell lymphomas [18, 19]. Chromosome 9p24.1 alterations range from low-level polysomy to near uniform and are reversely correlated with the survival of the cHL patients, and increased PD-L1 expression as the result of p24.1 amplification correlates with significantly shorter survival time [20, 21]. In addition, 9p24.1 amplification includes a novel regulatory loop of Janus kinase 2 (JAK2), and activation of the signaling of Janus kinase 2–signal transducers and activators of transcription (JAK2-STAT) further increase PD-1 ligand expression[10]. These findings therefore highlighted the superior therapeutic benefit of PD-1 blockade in cHL patients with 9p24.1 amplification [11, 20]. Additionally, recent study reported the potential value of PD-L1 amplification for the guidance of the immunotherapy in solid tumors, as six of nine patients (66.7%) with PD-L1 amplification had objective response to checkpoint blockade[22]. Previous study has focused on the mechanism of the changes in copy number of chromosome region in the CRC[23]. However, the PD-L1 gene status remains unknown in CRC patients.

A phase 2 study by *Le et al.* revealed that the dMMR subset of CRC is sensitive to the PD-1 blockade immunotherapy, and the underlying mechanism is mainly ascribed to microsatellite instability (MSI) [24, 25], which is associated with high tumor mutation burden, inflammatory microenvironment, and PD-L1 upregulation, the features generally sparse in pMMR CRC tumors. Although the response rate to anti-PD-1 therapy was significantly higher in dMMR vs. pMMR CRC patients, approximately 40% of the dMMR patients didn’t respond to anti-PD-1 therapy, and the reason remains unknown [24]. Unexpectedly, a recent case report presented a case of pMMR patient with PD-L1 gene amplification responding well to ICIs[26], further highlighted the potential clinical value of examining PD-L1 genetic alterations.

Approximately 15% of CRC patients harbor MSI, mainly due to loss of the DNA mismatch repair (MMR) protein MSH2, MSH6, MLH1, or PMS2[27]. Initially considered chromosomally stable, MSI tumors also displayed chromosomal alterations[27] and MSI-associated gene expression variation was ascribed to systematic DNA copy number aberrations[28]. To date, the precise incidence, nature, and clinical relevance of PD-L1 genomic alterations in the dMMR and pMMR CRC remain undefined. To characterize the PD-L1 gene alterations in CRC patients, we performed 9p24.1 FISH assay and IHC staining of PD-L1 expression on a cohort of 324 patients with newly diagnosed CRC and long-term outcome data. Our results identified low frequency but clinically relevant PD-L1 genetic alterations in both dMMR and pMMR CRC patients, and have implications in further sub-stratifying CRC patients for precision therapy options including immunotherapy.

## Materials and methods

The inclusion criteria for the study are as follows: (i) pathological evidence of adenocarcinoma CRC; (ii) complete baseline clinical information and laboratory data; (iii) formalin-fixed paraffin-embedded (FFPE) tumor samples; (iv) complete follow-up data. Patients with unknown microsatellite stability status were excluded. Patients had received radiotherapy prior to surgery were excluded since the treatment might influence PD-L1 alterations. However, in order to better analyze the PD-L1 genetic alteration status in colon cancer, especially the dMMR patients, we collected 160 consecutive dMMR patients between July 2011 and August 2013, and chose 164 pMMR patients from 1992 consecutive pMMR patients after matching the dMMR cohort with age, gender and TNM stage with the proportion of 1:1. Clinical stage was reclassified according to the eighth edition criteria of the American Joint Commission on Cancer/International Union Against Cancer (AJCC/UICC). Overall survival (OS) was defined as the time from the date of primary treatment to the date of death from any cause, or until the date of the last follow-up. Ethical approval was obtained from the institutions through the respective institutional review boards. The study protocol was designed in accordance with the guidelines outlined in the Declaration of Helsinki and was approved by the Ethics Committee of Sun Yat-sen University Cancer Center.

### Immunohistochemistry for MMR status and Ki67 proliferation index

MMR status was determined by IHC for MLH1, MSH6, MSH2, and PMS2. The following primary antibodies were used: anti-hMLH1 (ZSGB-BIO Corp, ZM-0154 (ES05) Beijing, China), anti-hMSH2 (ZSGB-BIO Corp, ZA-0622 (RED2), Beijing, China), anti-hMSH6 (ZSGB-BIO Corp, ZA-0541 (EP49), Beijing, China), and anti-hPMS2 (ZSGB-BIO Corp, ZA-0542 (EP51), Beijing, China). Tumor-infiltrating leukocytes were used as an internal control and the known dMMR CRC patients were used as external negative controls. MLH1/PMS2/MSH2/MSH6 negative protein expression was defined as tumor with loss of MLH1/PMS2/MSH2/MSH6 protein visualized by light microscopy. Tumors showing absolute absence of nuclear staining, with adjacent benign tissue showing nuclear staining, were considered as “loss of expression” [29].

Proliferation index of tumor cells was detected by the Ki67 primary Ab (AlexaFluor 488-conjugated, #11,882-D3B5; Cell Signaling Technology, Massachusetts, USA). Scores were classified based on the number of Ki 67^+^ nuclei per core. Only unequivocal nuclear staining was counted and the fraction of cells positively stained with anti-Ki67 is designated as the Ki67 fraction, or index.

### Immunohistochemistry for PD-L1, PD-1, and tumor-infiltrated immune cells

IHC staining was performed to assess PD-L1, PD-1, CD3, CD4, CD8, CD56 protein expression in the 324 human CRC cancer samples according to standard protocol [30]. Mouse monoclonal antibodies against CD3, CD4, CD8, CD56 protein (DAKO, Denmark) were used to test the expression of the corresponding protein. The rabbit monoclonal antibodies against anti-PD-L1 (anti-PD-L1: CST, #85,164-E1L3N) and PD-1 (anti-PD-1: CST, #55,789-D7D5W) were used for the analysis. IHC staining scores were performed separately for the tumor cells (TCs) and tumor-infiltrated immunocytes (ICs) by two independent observers (L.Y. and SH.L.), and following the Allred proportion score [31, 32]. A case was considered as “positive” if tumor cells were > 1% and immunocytes were > 1% for the PD-L1 score. For the PD-1 score, it was considered positive when the multiplied score was > 1. In addition, the degree of tumor-infiltrated lymphocytes was evaluated using 4-degree levels based on the visual assessment: 0 (no staining), 1 (5–10%), 2 (10–30%), 3 (30–40%), and 4 (40–100%). Sample slides with a score of 0 were regarded as negative and those with a score of 1, 2, or 3 were considered positive [7, 33].

### FISH for determination of PD-L1 copy number

PD-L1 copy number was investigated by fluorescence in situ hybridization (FISH) using the GSP PD-L1 Spectrum Orange/CSP 9 Spectrum Green probe (Guangzhou LBP Medicine Science and Technology Co., LTD., China) according to a published protocol with minor modifications [20]. FISH signals for each locus-specific probe were assessed under an Olympus BX51 TRF microscope (Olympus, Tokyo, Japan) equipped with a triple-pass filter (4’6’-diamidino-2-phenylindole [DAPI]/Green/Orange; Vysis, Abbott Molecular, Abbott Park, Ill, USA). PD-L1 copy number status was assessed by two independent observers, (F.W. and L.Y.), blinded to clinical or pathological characteristics. At least one hundred non-overlapping tumor cell nuclei were scored for the copy number of PD-L1. According the PD-L1 FISH criteria previously defined [34, 35], the PD-L1 gene status was classified into four groups as follows: (1) Disomy (normal copy number); (2) Deletion (target:control probe ratio of < 0.85); (3) Polysomy (more than five copies of the target probe and target:control probe ratio of < 2:1); and (4) Amplification (target: control probe ratio of ≥ 2:1 or the target probe ≥ 10). Polysomy, deletion, and gene amplification were considered as PD-L1 FISH+, and disomy was considered as PD-L1 FISH-. In each case, the average PD-L1 copy number was noted. Cases were graded by the highest observed level of PD-L1 alteration.

### Statistical analysis

Continuous variables were transformed into dichotomous variables at median value. Comparisons were performed using Fisher’s exact analysis for categorical variables. The Kaplan-Meier method was used for survival analysis by log rank tests. Variables achieving significance at the level of *P* < 0.05 were entered into multivariate Cox-regression analyses via stepwise procedures. Two-sided *P* < 0.05 was considered statistically significant, and Bonferroni correction for multiple comparisons were performed. Statistical data analyses were performed using software SPSS 22.0 (SPSS, Chicago, IL, USA).

## Results

### Spectrum and features of PD-L1 genetic alterations in CRC

**Fig. 1:**
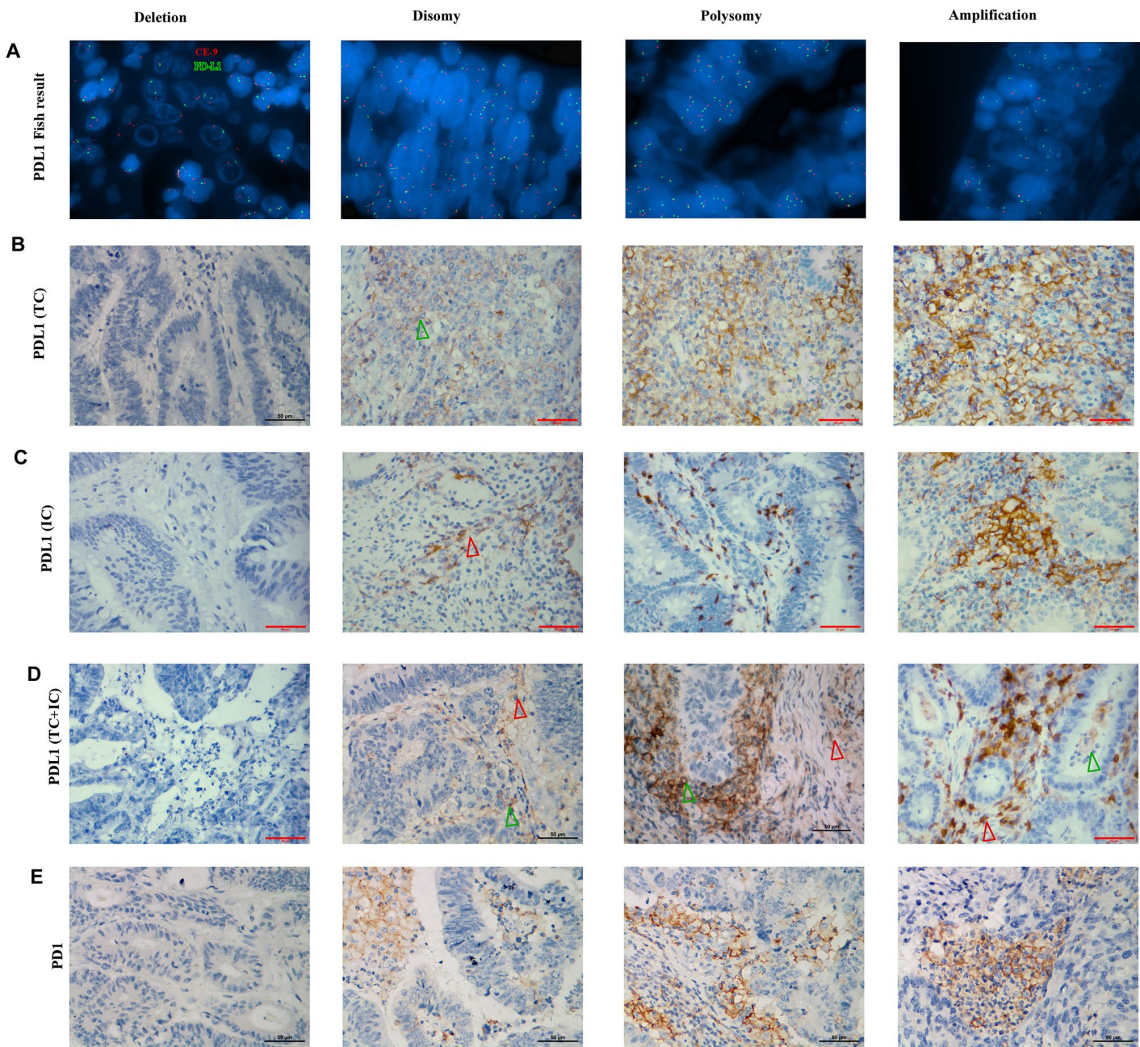
Genetic and immunohistochemical analyses of PD-L1 ligand expression. (A) Representative images of FISH results for the various categories. PD-L1 in Green (a), and centromeric probe (CEP9) in Red (b). (1) disomy (normal copy numbers); (2) Deletion (target:control probe ratio of < 0.85); (3) Polysomic (target:control probe ratio of 1:1, but more than five copies of the target probe); (4) Amplification (target:control probe ratio of > 2:1 or the target probe > 10). (B, C, D) PD-L1 immunohistochemistry in the colon cancer cases with PD-L1 deletion, disomy, polysomy, and amplification (tumor cells for B, immunocytes for C, tumor cells plus immonocytes for D); (E) PD-1 IHC in the same CRC cases. Scale bar = 50 μm

PD-L1 genetic alterations were tested by FISH in 324 CRC patients. Tumor cells were scored as deletion, disomy, polysomy, or amplification (representative images in Fig. 1A). There were 33 patients (10.2%) having aberrant PD-L1 genetic alterations, included 7 deletion (2.2%), 16 polysomy (4.9%), and 10 amplification (3.1%), and the remaining 291 cases were disomy (89.8%). Among the aberrant PD-L1 genetic alterations, 13(4.0%) cases were dMMR and 20 (6.2%) cases were pMMR. Of note, the 7 deletion cases (2.2%) were all in the dMMR cohort.

**Fig. 2:**
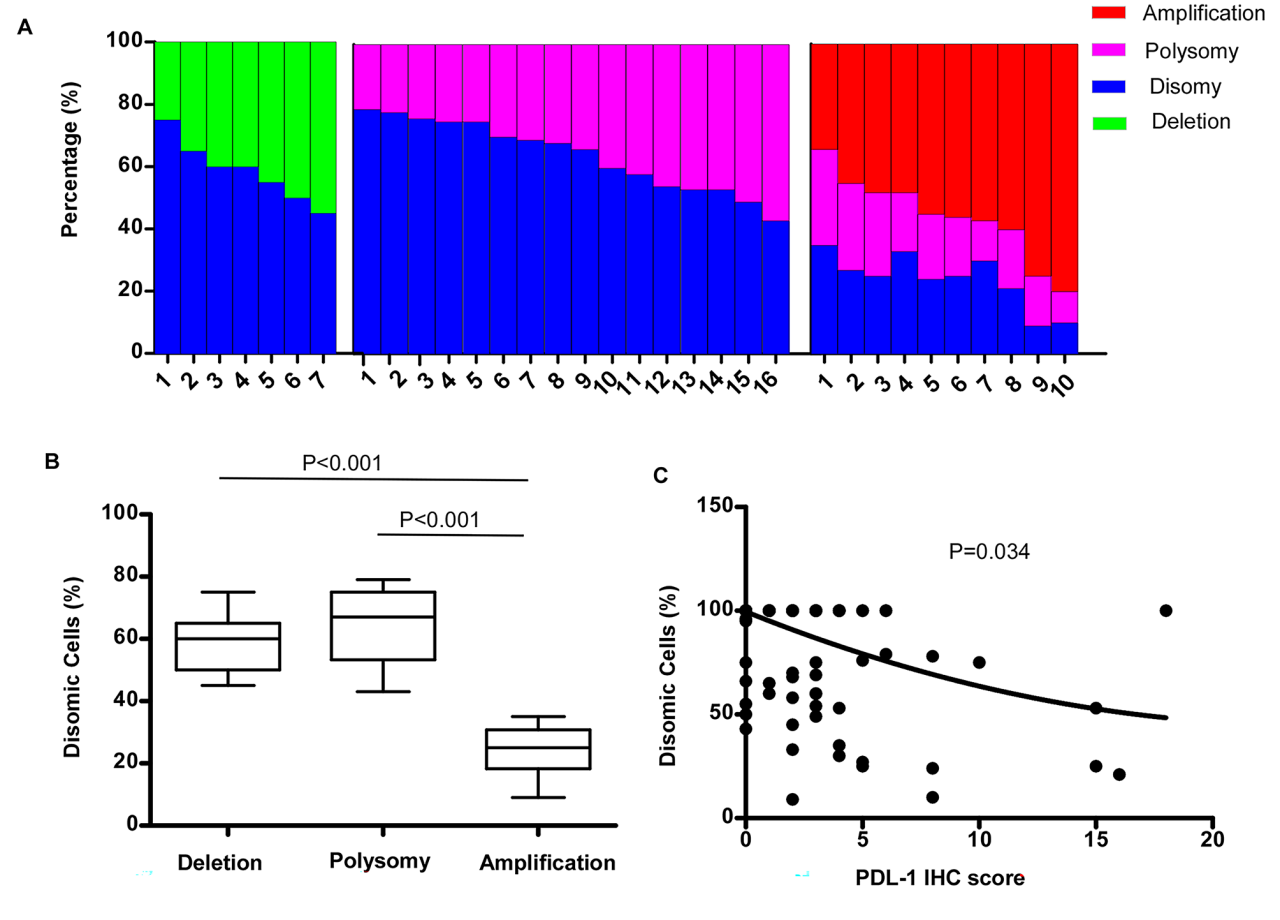
(A) The spectrum of 9p24.1 alterations in CRC. Individual tumors (deletion, polysomy, or amplification) are depicted as columns on the x-axis and the percentage of tumor cells with 9p24.1 disomy (blue), deletion (green), polysomy (light pink), and/or amplification (red) is shown on the y-axis. (B) Percentage of tumor cells with residual 9p24.1 disomy in CRC is classified by 9p24.1 alterations, as represented as box-and-whisker plots. CRC with 9p24.1 deletion, polysomy, and amplification have significantly different percentages of residual tumor cells with normal (disomic) 9p24.1 copy numbers (amplification vs. deletion, p < 0.001; amplification vs. polysomy, p < 0.001, Kruskal-Wallis test). (C) Association of PD-L1 protein expression and 9p24.1 copy number alterations. Residual 9p24.1 disomy is depicted on the Y-axis; PD-L1 immunohistochemistry and H-score (in quartiles) is shown on the X-axis. Quartiles are indicated by dashed lines. A highly significant decrease in percentage of residual 9p24.1 disomic cells in CRC with a higher PD-L1 IHC H-score is shown. p = 0.034, Kruskal-Wallis test

Although deletion, polysomy, and amplification were defined as aberrant genetic alterations, each of these samples still contained disomic cells to some degree. The residual disomic cell percentage range from 9 to 35% of cells for PD-L1 amplification, 43–78% of cells for PD-L1 polysomy, 45–75% for PD-L1 deletion. Besides, amplification also included 13–28% polysomic alteration. The percentage of residual PD-L1 disomic cells was highest in cases classified as polysomic alteration, intermediate in deletion alteration, and lowest in amplification alteration (amplification vs. deletion, p < 0.001; amplification vs. polysomy, p < 0.001; Kruskal-Wallis test; Fig. 2B).

The inverse association of PD-L1 alterations (amplification and polysomy) and residual disomy (Fig. 2A) pushed us to evaluate the correlation between residual PD-L1 disomy and the PD-L1 IHC immunostaining score, which was calculated as the percentage of the tumor cells with PD-L1 staining multiplied by its average intensity. As shown in Fig. 2C, there was apparent tendency of decreased residual PD-L1 disomy with elevated PD-L1 expression (p = 0.034, Kruskal-Wallis test; Fig. 2C).

### Association between PD-L1 genetic alterations and clinical/immunological characteristics

Age, gender, pathological types, tumor location, PLN (positive lymph node), TLN (total lymph node), TNM stage, MMR status, intestinal obstruction, Ki67 were chosen as the clinical characteristics and were compared among different PD-L1 genetic alteration groups. Patients with PD-L1 amplification tend to have a high incidence of PLN (*P* < 0.001); lack of intestinal obstruction (p = 0.010); pMMR status (p = 0.001); advanced N stage (p = 0.021); advanced stage (p = 0.001). The significance for PLN (p < 0.001), MMR status (p < 0.001), TNM stage (p = 0.02) remained after the adjustment for multiplicity (Supplementary Table 1). The correlation mostly remained when analysis only focused on disomy, polysomy and amplification.

The incidence of PD-L1 deletion and PD-L1 amplification increased by clinical stage (p = 0.001, 0% in stage I, 1.7% in stage II, 2.4% in stage III, and 14.3% in stage IV for PD-L1 deletion; 2.0% in stage I, 1.7% in stage II, 4.9% in stage III, and 14.3% in stage IV for PD-L1 amplification, Supplementary Fig. 1). By contrast, the incidence of PD-L1 disomy decreased by clinical stage (p = 0.001, 98.2% in stage I, 92.2% in stage II, 82.9% in stage III, and 71.4% in stage IV, Supplementary Fig. 1).

We next checked the concordance between the PD-L1 genetic alterations and the PD-L1 IHC staining intensity. For the 10 patients with PD-L1 amplification and the 7 patients with PD-L1 deletion, the PD-L1 IHC in TCs were all positive and all negative, respectively, for the 16 patients with PD-L1 polysomy, 12 patients were PD-L1 IHC positive in TCs (p < 0.001). The representative figures of the PD-L1 IHC were shown in Fig. 1. We then assessed the association of PD-L1 genetic alterations and tumor infiltrated lymphocytes (TILs). Among the 7 patients with PD-L1 deletion, all were positive for CD4 + T cells and CD8 + T cells as well as negative for CD68 + cells. Among the 26 samples with PD-L1 polysomy or amplification, the percentage of CD4 + T cells, CD8 + T cells and CD68 + cells positivity were 42.3%, 53.8% and 42.3%, respectively (p = 0.023 for CD4 + T cells, p = 0.048 for CD8 + T cells, and p = 0.005 for CD68 + cells), however, those significant correlations did not exist after adjustment for multiplicity (Table 1).

**Table 1:** Association between PD-L1 genetic alterations and immunological characteristics

Characteristics	All, Number (%)	Deletion Number (%)	Disomy, Number (%)	Polysomy, Number (%)	Amplification,Number (%)	P	Adjusted P	*P
**PD1**						0.257	5.14	
Negative	157(48.5%)	6 (85.7%)	138 (47.4%)	8 (50.0%)	5 (50.0%)			
Positive	167 (51.5%)	1 (14.3%)	153 (52.6%)	8 (50.0%)	5 (50.0%)			
**PDL1 (ICs)**						**< 0.001**	**< 0.001**	**< 0.001**
Negative	224 (69.1%)	3 (42.9%)	218 (74.9%)	3 (18.8%)	0 (0%)			
Positive	100 (30.9%)	4 (57.1%)	73 (25.1%)	13 (81.3%)	10 (100%)			
**PDL1 (TCs)**						**< 0.001**	**< 0.001**	**< 0.001**
Negative	258 (79.6%)	7 (100.0%)	248 (85.2%)	4 (18.8%)	0 (0%)			
Positive	66 (20.4%)	0 (0.0%)	43 (14.8%)	12 (81.3%)	10 (100%)			
**CD4 + T cell**						0.023	0.46	
Negative	142 (43.8%)	0 (0%)	127 (43.6%)	11 (68.8%)	4 (40.0%)			
Positive	182 (56.2%)	7 (100%)	164 (56.4%)	5 (31.3%)	6 (60.0%)			
**CD8 + T cell**						**0.048**	0.96	
Negative	165 (50.9%)	0 (0%)	152 (52.2%)	7 (43.8%)	5 (50.0%)			
Positive	159 (49.1%)	7 (100%)	139 (47.8%)	9 (56.3%)	5 (50.0%)			
**CD68**						**0.005**	0.1	**0.049**
Negative	166 (51.2%)	7 (100%)	152 (52.2%)	4 (25.0%)	3 (30.0%)			
Positive	158 (48.8%)	0 (0%)	139 (47.8%)	12 (75.0%)	7 (70.0%)			
**NK cell**						0.622	12.44	
Negative	308 (95.1%)	7 (100%)	276 (94.8%)	16 (100%)	9 (90%)			
Positive	16 (4.9%)	0 (0)	15 (5.2%)	0 (0%)	1 (10.0%)			

Abbreviations: *P value refer to the aasociation between PD-L1 genetic alterations (disomy, polysomy, amplification) and clinical/immunological characteristics in the cohort without the 7 PD-L1 deletion patients

Secondly, immunological characteristics were compared. In both of dMMR cohort and pMMR cohort, PD-L1 expression by IHC in TCs (both p < 0.001) and ICs (p = 0.048, p < 0.001, respectively) significantly correlated to PD-L1 genetic alterations. However, PD-1 expression by IHC only correlated to PD-L1 genetic alterations in dMMR cohort (p = 0.016). Meanwhile, the PD-L1 expression by IHC in PD-L1 amplification and the PD-L1 deletion were complete opposite, especially in TCs (Table 2). In dMMR cohort, the PD-L1 genetic alterations were significantly associated with CD8^+^ T cell (p = 0.032), CD4^+^ cell (p = 0.032), and CD68^+^ cells (p = 0.04). However, PD-L1 deletion was positive for CD8^+^ T cell and CD4^+^ cell, and negative for CD68^+^ cells, meanwhile, PD-L1 amplification was inverse to PD-L1 deletion, negative for CD8^+^ T cell and CD4^+^ cell, and positive for CD68^+^ cells (Table 2, the representative images in Supplementary Fig. 2). There was no association between PD-L1 genetic alterations and TILs (CD8, p = 0.172; CD56, p = 0.507; CD68, p = 0.383, and CD4, p = 0.182) in the pMMR cohort.

**Table 2:** Association between PD-L1 genetic alterations and clinical/immunological characteristics in dMMR cohort and pMMR cohort

Characteristics	dMMR							pMMR				
	Deletion	Disomy	Polysomy	Amplification	P	Adjusted P	*P	Disomy	Polysomy	Amplification	P	Adjusted P
**Stage**					0.020	0.16	0.046				0.189	1.512
I	0 (0%)	24 (16.3%)	0 (0%)	0 (0%)				24 (16.7%)	0 (0%))	1 (12.5%)		
II	3 (42.9%)	83 (56.5%)	2 (50%)	0 (0%)				82 (56.9%)	6 (50%)	3 (37.5%)		
III	2 (28.6%)	34 (23.1%)	2 (50%)	1 (50%)				34 (23.6%)	6 (50%)	3 (37.5%)		
IV	2 (28.6%)	6 (4.1%)	0 (0%)	1 (50%)				4 (2.9%)	0 (0%)	1 (12.5%)		
**PD1**					0.002	0.016	0.046				0.414	7.87
Negative	7 (100%)	98 (66.7%)	0 (0%)	0 (0%)				71 (49.3%)	8 (66.7%)	5 (62.5%)		
Positive	0 (0%)	49 (33.3%)	4 (100%)	2 (100%)				73 (50.7%)	4 (33.3%)	3 (37.5%)		
**PDL1 (ICs)**					0.006	0.048	0.071				< 0.001	< 0.001
Negative	3 (42.9%)	118 (80.3%)	3 (75.0%)	0 (0%)				100 (69.4%)	0 (0%)	0 (0%)		
Positive	4 (57.1%)	29 (19.7%)	1 (25%)	2 (100%)				44 (30.6%)	12 (100%)	8 (100%)		
**PDL1 (TCs)**					< 0.001	< 0.001	< 0.001				< 0.001	< 0.001
Negative	7 (100%)	121 (82.3%)	2 (50%)	0 (0%)				127 (88.2%)	3 (25.0%)	0 (0%)		
Positive	0 (0%)	26 (17.7%)	2 (50%)	2(100%)				17 (11.8%)	9 (75.0%)	8 (100%)		
**CD4 + T cell**					0.004	0.032	0.083				0.182	3.46
Negative	0 (0%)	79 (53.7%)	4 (100%)	2 (100%)				48 (33.3%)	7 (58.3%)	2 (25.0%)		
Positive	7 (100%)	68 (46.3%)	0 (0%)	0 (0%)				96 (66.7%)	5 (41.7%)	6 (75.0%)		
**CD8 + T cell**					0.004	0.032	0.064				0.172	3.27
Negative	0 (0%)	78 (53.1%)	4 (100%)	2 (100%)				74 (51.4%)	3 (25.0%)	3 (37.5%)		
Positive	7 (100%)	69 (46.9%)	0 (0%)	0 (0%)				70 (48.6%)	9 (75.0%)	5 (62.5%)		
**CD68**					0.005	0.040	0.012				0.383	7.28
Negative	7 (100%)	78 (53.1%)	0 (0%)	0 (0%)				74 (51.4%)	4 (25.0%)	3 (37.5%)		
Positive	0 (0%)	69 (46.9%)	4 (100%)	2 (100%)				70 (48.6%)	8 (75.0%)	5 (62.5%)		
**NK cell**					0.907	17.23					0.507	9.63
Negative	7 (100%)	141 (95.6%)	4 (100%)	2 (100%)				135 (93.8%)	12(100.%)	7 (87.5%)		
Positive	0 (0%)	6 (4.1%)	0 (0%)	0 (0%)				9 (6.3%)	0 (0%)	1 (12.5%)		

Abbreviations, *P value refer to the association between PD-L1 genetic alterations (disomy, polysomy, amplification) and clinical/immunological characteristics in the cohort without the 7 PD-L1 deletion patients

**Fig. 3:**
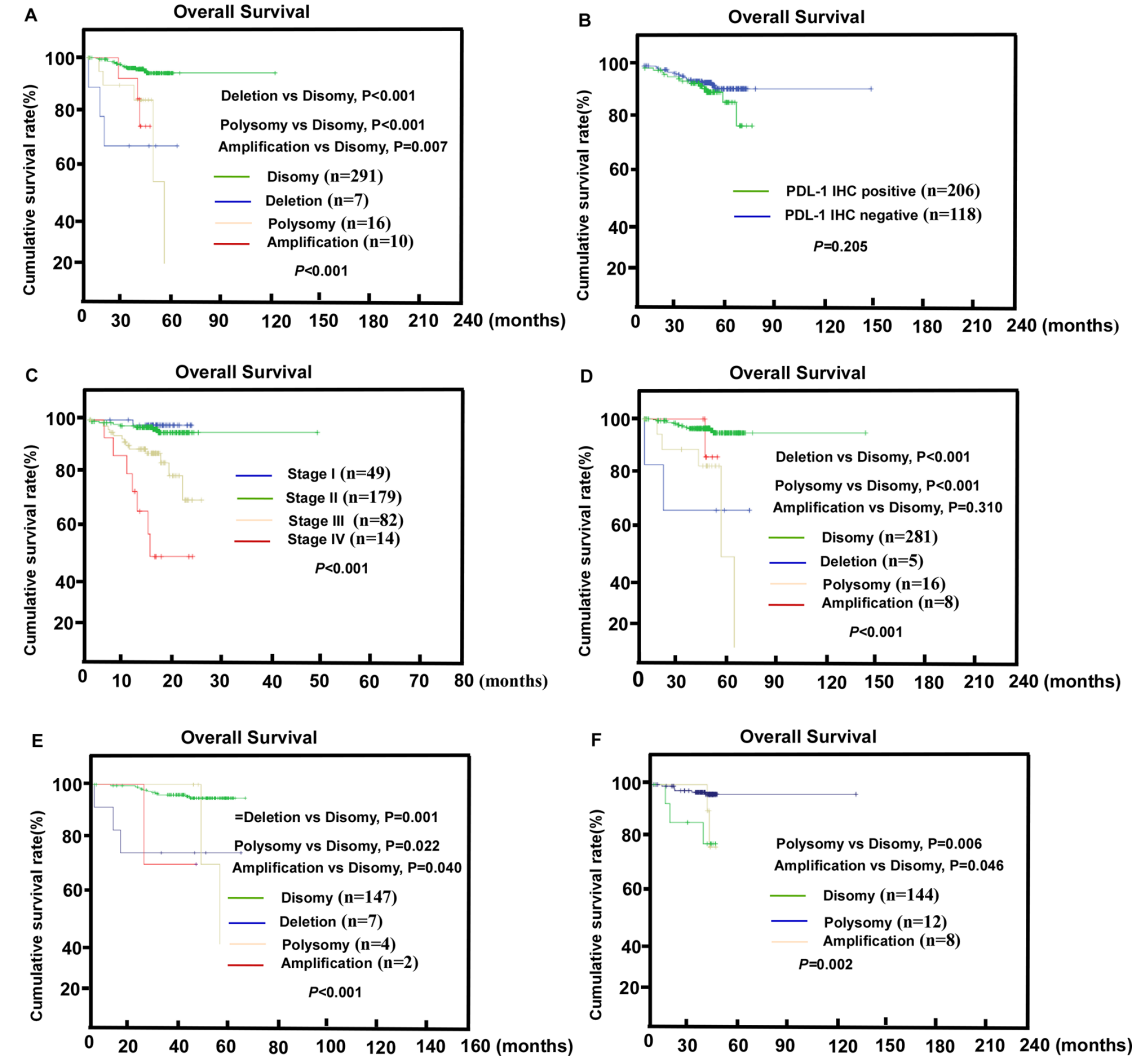
Clinical and genetic predictors for OS. (A) OS difference by 9p24.1 alterations in patients with colon-rectal cancer, log-rank test; (B), OS difference by PD-L1 IHC in patients with CRC, log-rank test; (C) OS difference by clinical stage in patients with CRC, log-rank test; (D) OS difference by 9p24.1 alterations in stage I + II + III, log-rank test; (E) OS difference by 9p24.1 alterations in dMMR cohort, log-rank test; (F) OS difference by 9p24.1 alterations in pMMR cohort, log-rank test

Thirdly, OS was compared among different groups. In both of dMMR cohort and pMMR cohort, aberrant PD-L1 genetic alterations had worse OS than patients with disomy (p < 0.001,p = 0.002, respectively. Figure 3E, F). However, the aberrant PD-L1 genetic alterations as an independent prognostic factor was only found in the dMMR cohort (p = 0.040, Supplementary Table 2). These results indicate that the PD-L1 genetic alterations may have greater prognostic significance in the dMMR cohort than in the pMMR cohort.

### Association between PD-L1 genetic alterations and overall survival

At the end of the study period (June 2018), 29 (8.9%) patients died. The 5-year Overall Survival (OS) for patients with PD-L1 disomy was 93.2%, higher than other three alterations respectively, PD-L1 deletion was 58.0% (p < 0.001), PD-L1 polysomy was 79.5%(p < 0.001), and PD-L1 amplification was 0% (p = 0.007) (Fig. 3A). At the same time, aberrant PD-L1 alterations was an independent prognostic factor in multivariate analysis (data not shown). However, there is no survival difference between the positive and negative expression of PD-L1 by IHC (the 5-year OS were 89.2% and 85.4% for PD-L1 IHC negative and PD-L1 IHC positive, respectively, p = 0.205, Fig. 3B). As expected, patients with advanced stage had a significantly worse outcome compared with patients in early stage (p < 0.001, log-rank test; Fig. 3C). Patients with aberrant PD-L1 genetic alterations in the early-stage (I + II + III) cohort have shorter OS than patients with PD-L1 disomy (p < 0.001, Fig. 3D, Amplification vs. Disomy, P = 0.310, this insignificance may result fromthe removal of the amplification cases of Stage IV). However, the survival difference was not observed in stage IV cohort (p = 0.307, Figure not showed), possibly due to only 14 patients in stage IV.

## Discussion

The high prevalence of PD-L1 gene amplification and its association with clinical features and response to anti-PD-1 therapy has been well documented in cHL, non-small cell lung cancer and melanoma [20, 36, 37]. However, the PD-L1 genetic alterations and its prognostic value by FISH assay in CRC patients have been sparsely investigated[22, 38]. In the current study, we found that the prevalence of aberrant PD-L1 genetic alteration was lower in CRC (10.2%) comparing to that in cHL, but much higher than 0.7% amplification rate (843 cases in 118,187 tumor samples from commercial database) reported in a recent study, which includes more than 100 types of solid tumor [38]. Such marked difference is likely due to different sample source and methods used for identifying genetic alterations. *Goodman et al.* used genomic profiling with FoundationOne assay covering maximum 405 cancer-related genes to study tumor samples from commercial database, and identified only 18 cases with PD-L1 amplification from total 9851 CRC samples. In our study, we have utilized FISH assay and PD-L1 IHC staining. It has been previously suggested that FISH may have better sensitivity and less technical difficulties for identifying specific genetic alterations than genomic sequencing on exon regions [39]. Furthermore, we have clinical information for all the samples, while less than 2% samples (2039/118,187) have clinical annotations. In another recent study, the authors also used FISH assay, and identified 2 samples with PD-L1 gene amplification from 13 PD-L1 positive samples by IHC staining, with 0 PD-L1 polysomy [38]. Thereby, we believe that the prevalence we identified from 324 CRC patients may be much closer to real situation, but further validation study with larger number of patients is warranted.

In our study, both deletion and amplification were associated with aggressive disease clinical features. Meanwhile, we found that as the PD-L1 gene copy number increased, meaning from disomy to polysomy to amplification, patients’ outcome became worse. Among the three aberrant alterations, from amplification to deletion to polysomy, the OS decreased by degree significantly, all worse than disomy. To our best knowledge, PD-L1 genetic deletion was for the first time detected in CRCs, and it was exclusively found in dMMR cohort; All samples with PD-L1 deletion have very distinct clinical features: they were all negative for PLN, PD-L1 expression in TCs, CD68 + cells and NK cells; but were all positive for CD4 + T cells and CD8 + T cells. Such distinct immune microenvironment features of PD-L1 genetic alterations in dMMR CRC patients’ warrants further study with larger populations, especially patients receiving anti-PD-1 treatment.

MSI-H/dMMR is the only FDA-approved genetic marker for anti-PD-1 therapy in CRC, though with low incidence[24, 25]. We then compared the PD-L1 genetic alterations in dMMR and pMMR separately. Many features of PD-L1 genetic alterations such as relation to clinical stages and OS that found in the whole cohort only exist in the dMMR cohort, which indicated that PD-L1 genetic alterations may impact more on dMMR cohort than pMMR. However, two striking features were found when dMMR and pMMR cohorts were analyzed separately. The first one is all PD-L1 deletions were in dMMR cohort, as PD-L1 expression was considered the major indicator for anti-PD-1 response, whether these patients still respond well to anti-PD-1 therapy? Nevertheless, about 40% dMMR CRC patients didn’t benefit from anti-PD-1 therapy, and the reason remains unknown [24]. Whether PD-L1 deletion contributes to such insensitivity needs further investigation. The second one is that PD-L1 amplification was mainly in pMMR cohort who had more positive PD-L1 expression in TCs and ICs; whether these patients would benefit from anti-PD-1 treatment is unknown. Interestingly, one recent report found that one colon cancer patient with MSS (pMMR) still benefited from immune checkpoint blockade treatment which likely due to PD-L1 amplification[37]. These data suggest that combining the PD-L1 genetic alterations and MMR status may be a more powerful biomarker for immune checkpoint blockade treatment for CRC patients. *Le et al.* had reported that MSI-H tumors have dense infiltration of lymphocytes[24] and the inhibitory status of the tumor microenvironment crucially determines the effectiveness of immunotherapy[40]. Additionally, we found that the correlation of PD-L1 genetic alteration with immune molecular features was mostly identified in dMMR cohort, which may also the cause of immune tolerance where tumor antigen escapes the appropriate immune response.

Though the key information came from the FISH assay and IHC results, the small amounts of patients randomly selected for whole exon sequencing validate the former two data is of reliability and compatibility. We are aware that the findings from one-center study and retrospective study have certain limitations and the conclusions drew needs further validation from independent studies. In addition, the implication of a few results remains un-explained such as the negative CD68 markers and NK markers in tumor tissues of samples with PD-L1 deletions; How would PD-L1 deletion affect macrophage or NK cell infiltration and alter the tumor microenvironment is unknown; Finally, the predictive value of PD-L1 genetic alterations in anti-PD-1 therapy efficacy warrants further study in CRC patients with anti-PD-1 therapy.

In summary, our data indicate the prevalence of PD-L1 genetic alterations was not common in CRC, but the aberrations were more frequently observed in the advanced stage, associated with more TILs infiltration, and with poorer overall survival, especially in dMMR cohort. Some immune molecular features were only observed in dMMR cohort. These findings need further validation and is worthy of exploring for the potential great value in the context of CRC cancer immunotherapy.

## Electronic supplementary material

Below is the link to the electronic supplementary material.

Supplementary Material 1

Supplementary Material 2

Supplementary Material 3

Supplementary Material 4

## Data Availability

Raw data was deposited in the Research Data Deposit (RDD, RDDB2018000508) system (http://www.researchdata.org.cn) of Sun Yat-sen University Cancer and can be obtained from the corresponding authors on reasonable request.

## Abbreviations

CRC: Colorectal cancer
MSS: Microsatellite stability
MSI: Microsatellite instability
FISH: Fluorescence in situ hybridization
dMMR: Mismatch repair deficient
pMMR: Mismatch repair proficient
OS: Overall survival
CTLA4: Cytotoxic T-lymphocyte antigen 4
PD-1: Programmed death 1
cHL: Classical Hodgkin Lymphoma
JAK: Janus Kinase 2
STAT: Signal transducers and activators of transcription
FFPE: Formalin-fixed paraffin-embedded
AJCC/UICC: American Joint Commission on Cancer/International Union Against Cancer
BAC: Bacterial artificial chromosome
IHC: Immunohistochemical
TCs: Tumor cells
ICs: Immune cells
TILs: Tumor infiltrated lymphocytes
PLN: Positive lymph node
APC: Antigen presenting cells
