# Supplementary material for: Characterisation of tumor microenvironment and prevalence of CD274/PD-L1 genetic alterations difference in colorectal Cancer
Source: BMC Cancer. 2023 Mar 9;23:221. doi: 10.1186/s12885-023-10610-1 (PMC9996909; doi:10.1186/s12885-023-10610-1)
Supplement: Supplementary file 1 — Supplementary Material 1 [file 12885_2023_10610_MOESM1_ESM.docx]

| supplementary Table 1. Association between PD-L1 genetic alterations and clinical characteristics | | | | | | | |
| --- | --- | --- | --- | --- | --- | --- | --- |
| **Characteristics** | **All,**  **Number (%)** | **Deletion**  **Number (%)** | **Disomy, Number (%)** | **Polysomy，**  **Number (%)** | **Amplification, Number (%)** | **P** | **Adjusted P** |
| **Age, years** |  |  |  |  |  | 0.621 | 12.42 |
| < 59 | 164 (50.6%) | 4 (57.1%) | 150 (51.5%) | 6 (37.5%) | 4 (40.0%) |  |  |
| ≥ 59 | 160 (49.4%) | 3 (42.9%) | 141 (48.5%) | 10 (62.5%) | 6 (60.0%) |  |  |
| **Sex** |  |  |  |  |  | 0.371 | 7.42 |
| Male | 125 (38.6%) | 3 (43.9%) | 108 (37.1%) | 8 (50.0%) | 6 (60.0%) |  |  |
| Female | 199 (61.4%) | 4 (57.1%) | 183 (62.9%) | 8 (50.0%) | 4 (40.0%) |  |  |
| **Pathology** |  |  |  |  |  | 0.625 | 12.5 |
| Well-differentiated | 6 (1.9%) | 0 (0%) | 6 (2.1%) | 0 (0%) | 0 (0%) |  |  |
| Moderately-differentiated | 267 (82.4%) | 6 (85.7%) | 236 (81.1%) | 16 (100%) | 9 (90.0%) |  |  |
| Low-differentiated | 51 (15.7%) | 1 (14.3%) | 49 (16.8%) | 0 (0%) | 1 (10.0%) |  |  |
| **Tumor location** |  |  |  |  |  | 0.975 | 19.5 |
| LCC | 186 (61.5%) | 4 (57.1%) | 166 (57.0%) | 10 (62.5%) | 6 (60.0%) |  |  |
| RCC | 138 (42.6%) | 3 (42.9%) | 125 (43.0%) | 6 (37.5%) | 4 (40.0%) |  |  |
| **TLN** |  |  |  |  |  | 0.440 | 8.8 |
| < 12 | 118 (36.4%) | 4 (57.1%) | 102 (35.1%) | 7 (43.8%) | 5 (50.0%) |  |  |
| ≥ 12 | 206 (63.6%) | 3 (42.9%) | 189 (64.9%) | 9 (56.3%) | 5 (50.0%) |  |  |
| **PLN*** |  |  |  |  |  | **<0.001** | **<0.001** |
| No | 241 (74.4%) | 7 (100%) | 223 (76.6%) | 8 (50.0%) | 3 (30.0%) |  |  |
| Yes | 83 (25.6%) | 0 (0%) | 68 (23.4%) | 8 (50.0%) | 7 (70.0%) |  |  |
| **Intestinal obstruction** |  |  |  |  |  | **0.010** | 0.2 |
| No | 274 (84.6%) | 3 (42.9%) | 247 (84.9%) | 14 (87.5%) | 10 (100%) |  |  |
| Yes | 50 (15.4%) | 4 (57.1%) | 44 (15.1%) | 2 (12.5%) | 0 (0%) |  |  |
| **MMR** |  |  |  |  |  | **0.001** | **<0.001** |
| dMMR | 160 (49.4%) | 7 (100%) | 147 (45.4%) | 4 (25.0%) | 2 (20.0%) |  |  |
| pMMR | 164 (50.6%) | 0 (0) | 144 (54.6%) | 12 (75.0%) | 8 (80.0%) |  |  |
| **TNM stage** |  |  |  |  |  | **0.001** | **0.02** |
| I | 49 (15.1%) | 0 (0) | 48 (16.5%) | 0 (0%) | 1 (10.0%) |  |  |
| II | 179 (55.2%) | 3 (42.9%) | 165 (56.7%) | 8 (50.0%) | 3 (30.0%) |  |  |
| III | 82 (25.3%) | 2 (28.6%) | 68 (23.4%) | 8 (50.0%) | 4 (40.0%) |  |  |
| IV | 14 (4.3%) | 2 (28.6%) | 10 (3.4%) | 0 (0%) | 2 (20.0%) |  |  |
| **Ki67 index** |  |  |  |  |  | 0.929 | 18.58 |
| NA | 8 (2.5%) | 0 (0%) | 7 (2.4%) | 1 (6.3%) | 0 (0%) |  |  |
| <60% | 164 (50.6%) | 4 (57.1%) | 147 (50.5%) | 7 (43.8%) | 6 (60.0%) |  |  |
| >60% | 152 (46.9%) | 3 (42.9%) | 137 (47.1%) | 8 (50.0%) | 4 (40.0%) |  |  |

Abbreviations: CRC, colorectal cancer; Well-differentiated, Well-differentiated adenocarcinoma; Moderately-differentiated, Moderately-differentiated adenocarcinoma; Low-differentiated, Low-differentiated adenocarcinoma; LCC, Left sided colon cancer; RCC, right sided colon cancer; MMR, Mismatch repair; dMMR, mismatch repair deficient; pMMR, mismatch repair proficient; TLN, Total lymph node cleared in surgery; PLN, positive lymph node; *，2 of the 7 people of PDL1 deletion is stage III, with the negative positive lymph nodes since merely having tumor cultivation nodule and fibrous tissue infiltration;

Supplementary Table 2. Multivariate analysis of the dMMR cohort and the pMMR cohort

| Characteristics | dMMR | | | pMMR | | |
| --- | --- | --- | --- | --- | --- | --- |
|  | HR | 95%CI | P | HR | 95%CI | P |
| Intestinal obstruction, (No vs Yse) | 3.734 | 1.091-12.785 | 0.036 | NA | NA | NA |
| N stage |  |  | 0.006 | NA | NA | NA |
| 0 | 1.000 | 1.000 |  | NA | NA | NA |
| 1 | 2.250 | 0.234-21.631 | 0.482 | NA | NA | NA |
| 2 | 6.151 | 2.004-18.886 | 0.002 | NA | NA | NA |
| PDL1 (FISH) |  |  | 0.040 |  |  | 0.673 |
| Disomy | 1.000 | 1.000 |  | 1.000 | 1.000 |  |
| Deletion | 5.994 | 1.500-23.947 | 0.011 | 1.719 | 0.433-6.819 | 0.441 |
| Polysomy | 2.428 | 0.652-9.040 | 0.186 | 2.103 | 0.211-20.935 | 0.526 |
| Amplification | 4.787 | 0.907-25.278 | 0.065 |  |  |  |
| Distant metastasis or relapse, (No vs Yes) | NA | NA | NA | 7.847 | 1.824-33.764 | 0.006 |

Abbreviations: dMMR, mismatch repair deficient; pMMR, mismatch repair proficient
