# Supplementary figures and images for: Characterisation of tumor microenvironment and prevalence of CD274/PD-L1 genetic alterations difference in colorectal Cancer
Source: BMC Cancer. 2023 Mar 9;23:221. doi: 10.1186/s12885-023-10610-1 (PMC9996909; doi:10.1186/s12885-023-10610-1)

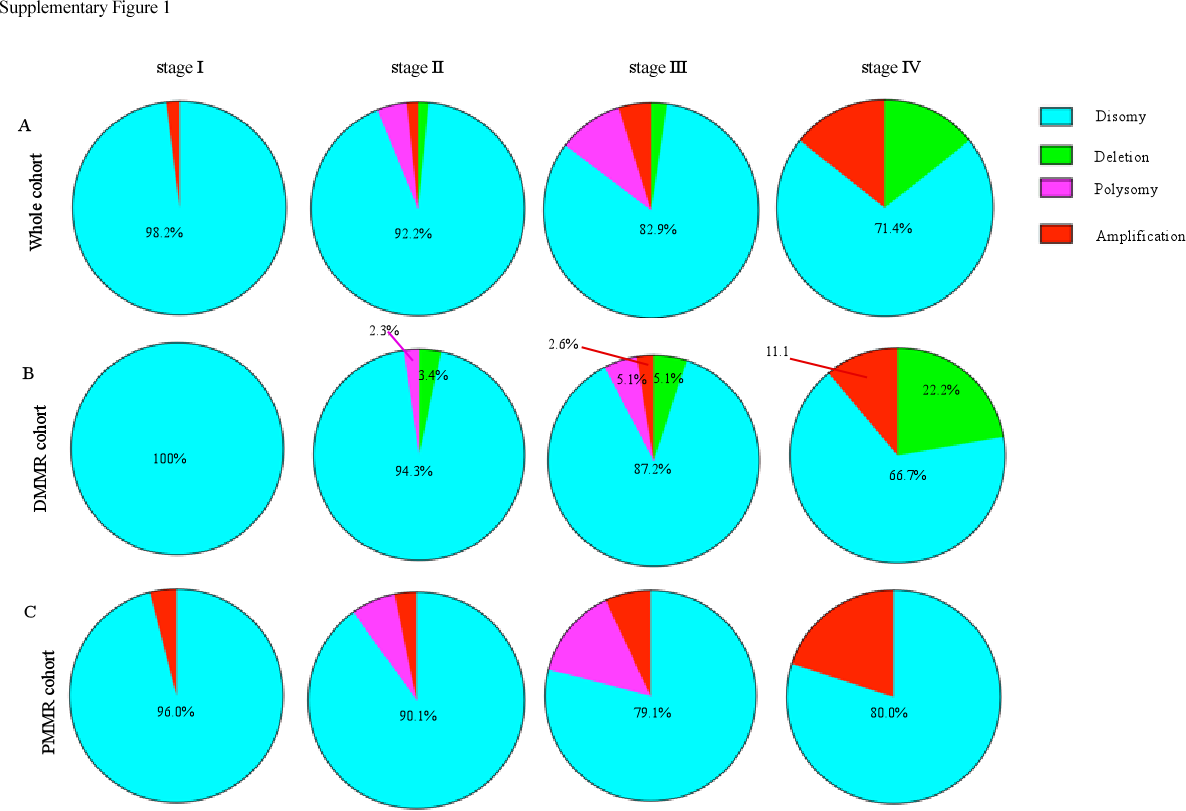

Supplement: Supplementary file 2 — Supplementary Material 2 [file 12885_2023_10610_MOESM2_ESM.tif]

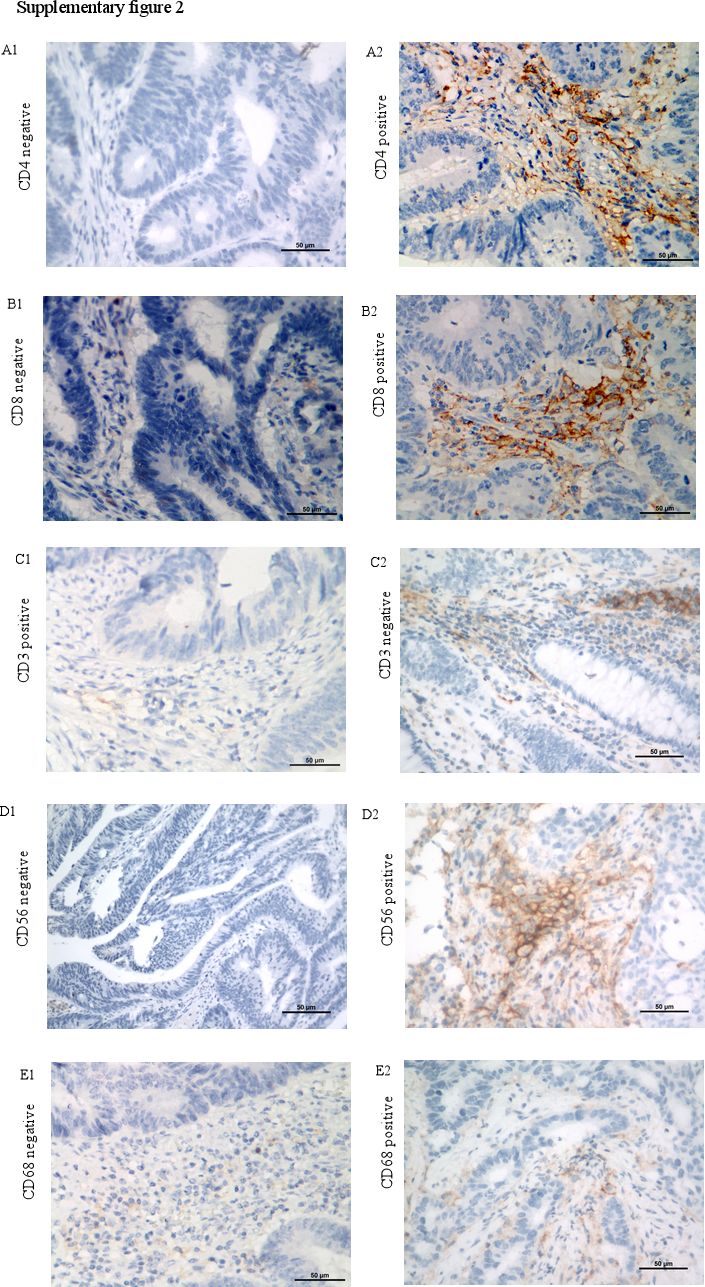

Supplement: Supplementary file 3 — Supplementary Material 3 [file 12885_2023_10610_MOESM3_ESM.tif]
