# Supplementary material for: Characterisation of tumor microenvironment and prevalence of CD274/PD-L1 genetic alterations difference in colorectal Cancer
Source: BMC Cancer. 2023 Mar 9;23:221. doi: 10.1186/s12885-023-10610-1 (PMC9996909; doi:10.1186/s12885-023-10610-1)
Supplement: Supplementary file 4 — Supplementary Material 4 [file 12885_2023_10610_MOESM4_ESM.docx]

Supplementary Figure 1. Frequency of PDL-1 alterations (deletion, disomy, polysomy, amplification) by clinical stage: A (all cohort), B (dMMR cohort), or C (pMMR cohort). The incidence of PDL-1 amplification is significantly different in clinically staged patients (Kruskal-Wallis test).

Supplementary Figure 2. Tumor associated PD-L1 negative immune cells (A) stained positive for CD4+ T cells (B), CD8+ T cells (C) and negative for CD68+ cells(D). Tumor associated PD-L1 positive immune cells (E) stained negative for CD4+ T cells (F), CD8+ T cells (G) and positive for CD68+ cells(H).
